# Pathophysiology and Management Strategies for Post-Stroke Spasticity: An Update Review

**DOI:** 10.3390/ijms26010406

**Published:** 2025-01-05

**Authors:** Bei Chen, Tong Yang, Zi Liao, Feiyue Sun, Zhigang Mei, Wenli Zhang

**Affiliations:** 1 Key Laboratory of Hunan Province for Integrated Traditional Chinese and Western Medicine on Prevention and Treatment of Cardio-Cerebral Diseases, College of Integrated Traditional Chinese Medicine and Western Medicine, Hunan University of Chinese Medicine, Changsha 410208, China; 20232130@stu.hnucm.edu.cn (B.C.); 20232122@stu.hnucm.edu.cn (T.Y.); liaozizizi@stu.hnucm.edu.cn (Z.L.); sunfeiyue@stu.hnucm.edu.cn (F.S.); 2 School of Pharmacy, Hunan University of Chinese Medicine, Changsha 410208, China

**Keywords:** stroke, spasticity, pathophysiology, intervention

## Abstract

Post-stroke spasticity (PSS), characterized by a velocity-dependent increase in muscle tone and exaggerated reflexes, affects a significant portion of stroke patients and presents a substantial obstacle to post-stroke rehabilitation. Effective management and treatment for PSS remains a significant clinical challenge in the interdisciplinary aspect depending on the understanding of its etiologies and pathophysiology. We systematically review the relevant literature and provide the main pathogenic hypotheses: alterations in the balance of excitatory and inhibitory inputs to the descending pathway or the spinal circuit, which are secondary to cortical and subcortical ischemic or hemorrhagic injury, lead to disinhibition of the stretch reflex and increased muscle tone. Prolongation of motoneuron responses to synaptic excitation by persistent inward currents and secondary changes in muscle contribute to hypertonia. The guidelines for PSS treatment advocate for a variety of therapeutic approaches, yet they are hindered by constraints such as dose-dependent adverse effects, high cost, and limited therapeutic efficacy. Taken together, we highlight key processes of PSS pathophysiology and summarize many interventions, including neuroprotective agents, gene therapy, targeted therapy, physiotherapy, NexTGen therapy and complementary and alternative medicine. We aim to confer additional clinical benefits to patients and lay the foundation for the development of new potential therapies against PSS.

## 1. Introduction

Spasticity is one of the most common motor defects secondary to stroke, which significantly affects post-stroke disability and daily life activities [1]. The duration, localization, and the number and size of infarcts are strongly associated with the risk of post-stroke spasticity (PSS) impairment [2]. Recently, the concept of spasticity has been extensively investigated. Spasticity is still widely defined as a velocity-dependent increase in muscle tension, presenting as involuntary muscle activation, mainly due to limb motor and sensory disorders caused by changes in the upper motor neurons [3]. A comprehensive review of the literature revealed that the incidence of spasticity following acute stroke ranges from 19% to 26.7%, and after recurrent stroke, it ranges from 17% to 42.6% [4]. In the chronic phase, spasticity affects up to 97% of stroke survivors with moderate-to-severe motor deficits [5]. While the onset of spasticity varies widely, it typically manifests between 1 and 6 weeks after injury, reaching peak severity within 1–3 months after stroke [6]. The elevated prevalence and persistence of spasticity necessitate an investigation of its pathophysiology and the development of an appropriate treatment approach.

Spasticity, as a major medical problem following stroke, occurs due to local activation of the muscle spindle; however, the propagation and manifestation of spasticity require the involvement of the central nervous system. Although the pathophysiology of PSS is not yet fully understood, there is growing evidence that spasticity occurs due to changes in supraspinal origins, spinal interneuronal networks, and peripheral muscles, leading to stretch reflex hyperexcitability. This is characterized by muscle overactivity and exaggerated reflex responses to peripheral stimuli. It has been revealed that pharmacological treatment, including oral and injectable forms, can affect the motor control pathway and peripheral muscles [7]. Currently, approved oral antispastic medications show limited therapeutic effect in reducing generalized spasticity and probably cause drowsiness and tiredness [8]. Although a botulinum toxin injection is the preferred treatment for PSS, its cost-effectiveness has not been proven [9]. Nonpharmacological and pharmacological treatments are often used in combination to control spasticity. In summary, all these treatments have some limitations, restricting their clinical application, and few therapeutic agents are available to improve patient outcomes. Furthermore, direct effects like increased muscle tone and indirect effects like daily activities contribute to challenges in developing pharmacological treatments for PSS. Current studies require controlled protocols to determine management strategies and precise treatment options for PSS. The main pathogenic hypotheses of spasticity and the importance of assessment, as well as multidisciplinary approaches to spasticity treatment, are reviewed in this article.

## 2. Methods

The search strategy involved conducting a computer-based online search of the Cochrane Library, Embase, Medline, PubMed, and Web of Science databases to retrieve articles published. To enhance both the specificity and sensitivity of the search, a combination of specific text words and MeSH terms was utilized. These terms encompassed “motor cortex”, “motor neurons”, “afferent neurons”, “efferent neurons”, “spastic”, “muscle spasticity”, “stroke”, “ischemic stroke”, and “hemorrhagic stroke”. The retrieved results were then refined by carefully screening the titles and abstracts, with a particular focus on studies that delved into the phenomena or mechanisms underlying PSS. No language restrictions were applied during the search process. The review integrates and synthesizes the findings from the selected articles, offering a comprehensive overview of PSS and providing valuable insights into potential therapeutic applications based on this research field.

## 3. The Anatomy of Descending Motor Conduction Pathways

**Figure 1:**
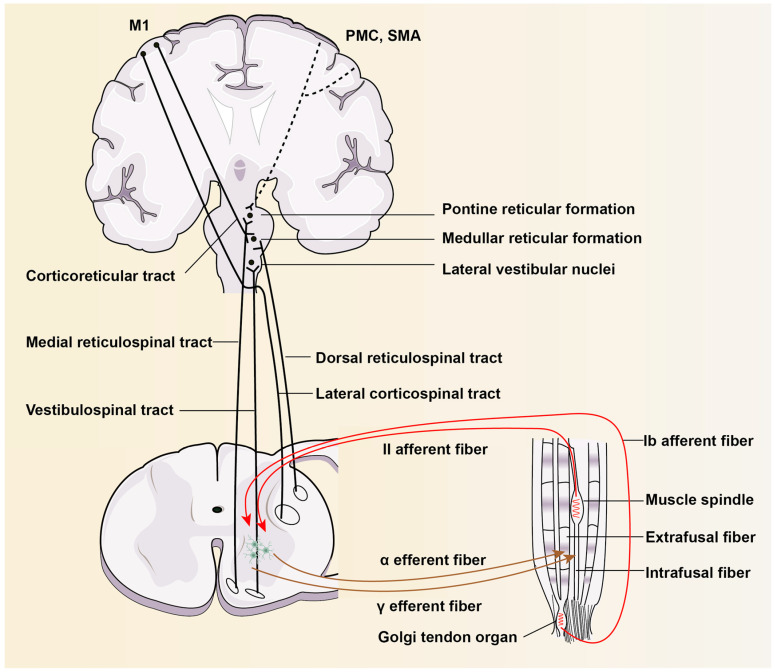
The anatomy of descending motor conduction pathways. The spinal cord, as the final common motor pathway, transmits signals between the brain and muscle. Spinal reflexes are coordinately controlled by peripheral afferents and central motor commands. (Red arrow): Afferent nerve; (Brown arrow): Efferent nerve.

The skeletomotor system is primarily based on a projection from the precentral frontal lobe back to the skeletal muscle of the brain, which involves the cortical and subcortical centers, brainstem, spinal cord, and muscle, with higher centers modulating lower centers (Figure 1).

It is generally accepted that the primary motor cortex (M1), premotor cortex (PM), and supplementary cortex areas (SMAs) are directly involved in the control of movement. The cerebral cortical motor area can stimulate motor neurons and interneurons directly through the brainstem via the corticospinal tract (CST), which is primarily involved in voluntary movements. The dorsal cortico-reticulo-spinal tract (CRST) receives input from the contralateral primary motor cortex (M1) and provides inhibitory downward outputs to spinal motor circuits [10]. The medial reticulospinal tract (RST) originates primarily from the pontine tegmentum and is facilitated by the ipsilateral premotor cortex (PMC) and supplementary cortex area (SMA) through the corticoreticular pathway. The medial CRST sends excitatory outputs to spinal motoneurons. The vestibulospinal tract (VST) originates from the lateral vestibular nucleus, descends virtually uncrossed, and controls extensors rather than flexors. The realization of movement, whether reflex or random, requires the contraction of innervated muscles through the excitation of the spinal motor neurons [11]. Spinal reflexes are coordinately controlled by sensory feedback to the spinal cord from the muscle and central motor commands. The extrafusal muscle fibers of skeletal muscle are the key contractile fibers that are innervated by the axons of α motor neurons, whereas the intrafusal muscle fibers are innervated by γ motor neurons. Positional and force commands are transmitted from the brain to the spinal cord, and α motor fibers are individually stimulated, while γ fibers are stimulated and α-γ coactivation occurs, resulting in the contraction of extrafusal and intrafusal fibers.

## 4. Pathophysiology of PSS

**Figure 2:**
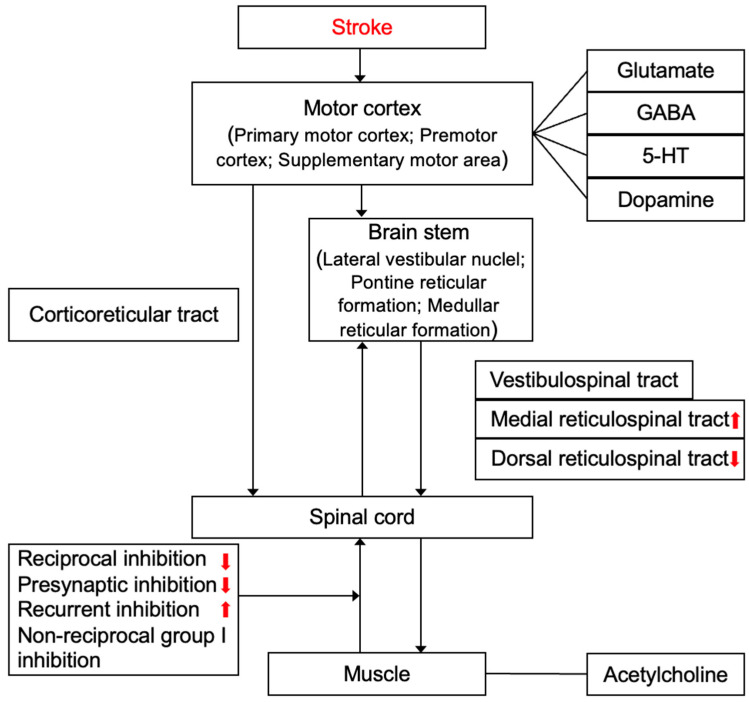
Flowchart of pathophysiological mechanisms underlying post-stroke spasticity. (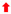): Increased; (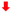): Decreased.

**Figure 3:**
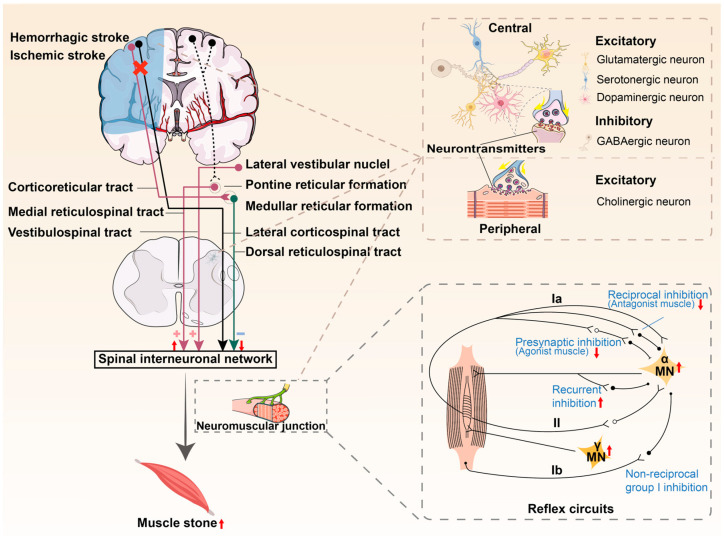
Pathophysiology of post-stroke spasticity (PSS). A stroke on one hemisphere, resulting in damage to both the CST and CRST, causes a decrease in output signals. The cortico-medial reticulospinal tracts (RST) of the contralateral cerebral hemisphere exhibit increased excitability and lack inhibition from the dorsal RST of the ipsilateral side, resulting in hyperexcitability or spontaneous firing in spinal motor neurons. (+): Excitatory; (−): Inhibitory;(×): Impaired; (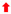): Increased; (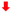): Decreased.

Stroke can cause injuries to upper motor neurons, located in the motor areas of the cerebral cortex, and control the transmission of motor signals to the spinal cord. It induces hyperactive reflexes in lower motor neurons and increased muscle due to an imbalance in the excitatory and inhibitory regulation of reflexes transmitted downward to the spinal tracts (Figure 2 and Figure 3).

### 4.1. Cortical and Subcortical Alterations

Stroke patients exhibit significant heterogeneity for stroke localization (cortical and subcortical), types (ischemic stroke and hemorrhagic stroke), and the presence of concomitant vascular injuries.

The imbalance between brain metabolic capacity and transport as well as the distribution of oxygen and glucose during stroke ultimately results in a complex combination of processes. Various mechanisms, including excitotoxicity, mitochondrial death pathways, the release of free radicals, protein misfolding, apoptosis, necrosis, autophagy, and inflammation, either independently or in conjunction, ultimately result in cell death [12]. Long-term synaptic transmission deficits were detected in the sensorimotor cortex after transient ischemia in rats [13]. Spasticity occurs in cortical lesions due to the associated involvement of cortical reticular fibers and the connection between the PM. A strong correlation was observed between the activity in the contralateral cortical areas and spasticity status after stroke [14]. However, activation in the ipsilesional sensorimotor cortex is correlated with the degree of stroke damage in the brain. Cortical and subcortical lesions indicate a higher risk of PSS development than cortical lesions alone [15]. Another study demonstrated that the superior corona radiata, internal capsule posterior limb, posterior corona radiata, thalamus, putamen, PM, and insula are associated with upper-limb spasticity. Conversely, the superior corona radiata, internal capsule posterior limb, caudate nucleus, posterior corona radiata, thalamus, putamen, and external capsule are associated with lower-limb spasticity [16]. The putamen located at lenticulostriate branches is a “spastic” region, not just a common stroke site, and has been identified as the region that distinguishes stroke survivors with and without spasticity [17].

### 4.2. Imbalanced Descending Supraspinal Regulations

Normal activity of the spinal stretch reflex pathway is primarily regulated by excitatory and inhibitory downward signals in the spinal cord and occurs at various points in the reflex pathway. Muscle tone is modulated by inhibitory signals from the dorsal RST and facilitated by influences from the medial RST and VST. Brain injury following a stroke may affect CST integrity and conduction function. In strokes with cortical and internal capsule damage, the CST and corticoreticular tract are often injured due to their anatomical proximity, causing a loss of cortical eutrophication inputs to medullary inhibitory centers. However, the involvement of the CST alone is insufficient to produce spasticity [18]. Although VST is responsible for decerebrate rigidity, it has a limited role in spasticity [19]. Findings from animal studies and advanced neuroimaging in humans do not support VST directly causing spasticity; however, VST may influence spasticity through the VST-RST connection [20]. The medial RST becomes unopposed and gradually hyperexcitable without the inhibitory influence of the dorsal RST from this center following stroke, providing excitatory inputs to the spinal motor neurons [21]. An in vivo study demonstrated that hyperexcitability of the RST in the resting state occurs during the spastic phase rather than the non-spastic phase of relaxation or recovery [22]. It is widely accepted that PSS is closely associated with RST hyperexcitability and its antigravity effects [23].

### 4.3. Changes in the Spinal Cord

The neuromuscular junction translates electrical impulses generated by motor neurons into electrical activity in muscle fibers. The balance between spinal motor neuron inhibition and excitation input changes, leading to spasticity and loss of joint mobility and dysfunction.

#### 4.3.1. Reflex Circuits

Random limb movements are initiated in the brain, but the neurons that are responsible for activating the muscles (motor and interneurons) are located in the spinal cord [24]. Variability in connections between muscle spindle afferents and spinal neurons can be balanced by varying the influence of these afferents on spinal target cells. Excessively strong actions of afferents can be moderated by presynaptic inhibition or inhibitory interneurons [25]. Spinal reflex pathways may modify monosynaptic excitatory actions, which can contribute to the tonic components of the stretch reflex. These reflex circuits include presynaptic inhibition of Ia afferent terminals, reciprocal inhibition of the muscle spindle, nonreciprocal group I inhibition of the Golgi tendon organ, recurrent inhibition, and presynaptic inhibition of muscle spindle group II afferent nerves from antagonistic muscles.

Activation of this presynaptic inhibitory circuit decreases neurotransmitter release in the synaptic gap between the presynaptic terminals of Ia and the membranes of α motor neurons, leading to presynaptic inhibition. The presynaptic inhibition in the upper limbs was significantly reduced in stroke populations; however, this reduction was not unique to the affected side [26]. Moreover, reduced presynaptic inhibition is not a causative factor for spasticity in resting hemiplegic patients [27]. Ia afferent fibers have direct synaptic contact with motoneurons projecting to layer XI of the gray matter of the spinal cord to generate excitatory postsynaptic potentials, while double synaptic contact occurs with interneurons projecting to layer VII of the gray matter to generate inhibitory postsynaptic potentials. Ia reciprocal inhibition was reduced in patients with post-stroke upper-limb spasticity [28]. It has been shown that reduced reciprocal inhibition may play a major role in the pathophysiology of spasticity, which is true for autonomous and reflex activities [29]. Commissural interneurons, activated by reticulospinal neurons, influence motoneurons directly and by modulating premotor interneurons in group Ib and II afferent pathways. They affect both excitatory and inhibitory premotor interneurons, aiding in selecting reflex actions during centrally initiated movements [30]. Nonreciprocal group I inhibition is caused by the activation of Ib afferents from the Golgi tendon organ and is mediated by segmental interneurons. Although this inhibition was easily demonstrated in healthy subjects, it failed to produce any inhibition on the paralyzed side in hemiplegic patients and has been replaced by facilitation in stroke patients [31]. This facilitation may be attributed to enhanced oligosynaptic group I facilitation superimposed on invariant Ib inhibition, or to reflex reversal between alternative Ib inhibition and Ib facilitation pathways, as normally occurs during movement [32]. Patients with spasticity, especially those with gait disorders, lack modulation of Ib inhibition during tetanic antagonist contractions [33]. This observation suggests that changes in Ib inhibition play a role in the pathophysiology of spasticity. Patients exhibit increased recurrent inhibition at rest after stroke, presumably due to the release of Renshaw cells from the descending inhibitory control. They receive excitatory collaterals from motor axons and project back to motor neurons and Ia inhibitory interneurons. It has been proven that, one month after a peripheral botulinum toxin type A (BoNT-A) injection, the resolution of PSS may be linked to the inhibition of spinal cord relapse inhibitory pathway activity, which may occur through axonal transport and blockade of cholinergic synapses between the complex collateral branches of motor axons and Renshaw cells [34]. The increased excitability of the II fibers in the quadriceps femoris motoneurons can cause spasticity after stroke [35]. However, there is no significant correlation between the degree of facilitation of II fibers and the degree of spasticity [36].

#### 4.3.2. Intrinsic Properties of the Motoneuron

Motor neurons are nerve cells with cell bodies located in the ventral horn of the spinal cord; their fibers project outside the spinal cord to control effector organs. Motor neurons in stroke survivors exhibit hyperexcitability and muscles tend to exhibit prolonged spontaneous discharges of motor units. An enhanced tendency to evoke PICs is frequently generated by increased subthreshold depolarization of motor neurons or increased monoaminergic input from the brainstem, expanding inputs to motor neurons and inducing discharges that cause motor neurons to become hyperexcited to inputs and allow transient inputs to initiate long-lasting self-sustained firing [27,37]. Depolarization of a membrane potential is maintained by intrinsic properties, even if the stimulus that triggered it has terminated. It has been suggested that monoaminergic facilitation of contralateral motor neurons may have a direct effect on the development of PSS [38]. The monoamines NE and 5-HT indirectly modulate the state of voltage-gated CaV1.3 channels located in the dendrites of alpha motor neurons, significantly increasing spinal motor neuron excitability. Moreover, Gorassini et al. expressed that plateau potentials are activated during spasticity and appear to contribute to spasticity [39]. The voltage-dependent and sustained inward calcium and sodium currents are particularly relevant because they amplify and prolong the motor neuron’s response to synaptic excitation. However, it has been demonstrated that spontaneous firing of motor units in spastic stroke survivors, in contrast to the largely PIC-based mechanism proposed for patients with spinal cord injury, may be partially attributable to a tonic low-level ionotropic drive of the motoneuron pool [40]. This maintains a certain proportion of motoneurons close to the firing threshold and thus it is more readily activated at rest.

### 4.4. Secondary Changes in Muscle

Spasticity is not only associated with increased muscle tone resulting from neural-mediated hyperreflexia but may also be associated with alterations in muscle mechanical properties. Changes in muscle mechanics, collagen tissue, and tendon properties occur gradually, leading to increased muscle spasticity [41], which may be adaptive and secondary to paresis. Changes in motor units and their contractile properties may be adaptive to changes in the intrinsic properties of the muscle and axial transfer of motion. When paralysis occurs after a stroke, the muscles remain in a shortened position, and the muscle fibers are twice as stiff as in normal subjects to produce optimal force on the shortened muscles [42]. This partially compensates for paralysis and allows functional movement at a simpler level of organization. Soft tissue alterations may cause a faster transmission of traction to the muscle spindle, which can increase sensory input to the spindle and spasticity. Conversely, increased spindle sensitivity after stroke increases peripheral afferent inputs to the spinal motor neuron, setting the stage for over-sensitization of the stretch [43]. Muscle fibers in the muscle spindle and Golgi tendon organ collaborate to regulate muscle control and contraction, resulting in muscle tone. Pulling on the muscle bundles increases the rate at which motor neurons release impulses. However, Pandyan et al. proposed that spasticity is a sensory–motor control disorder, rather than simply a phenomenon of increased resistance or impaired movement in a joint or limb [44]. Patients who have developed spasticity three months after a stroke are thought to develop this behavior due to intrinsic muscle changes. This was thought to be a functional spastic movement disorder, which is different from spasticity. Therefore, secondary muscle changes can induce hypertonia; however, the effect on spasticity remains unclear.

### 4.5. Neurotransmitters

Although studies specifically addressing the role of neurotransmitters in PSS are limited, several neurotransmitters, including amino acids, cholines, and monoamines, regulate muscle tone and function. A decrease in the release of inhibitory neurotransmitters or an increase in excitatory inputs disrupts this balance, resulting in spasticity. The cause of the decrease in the excitation–inhibition balance after a stroke remains unknown. Glutamate (Glu) has been described as one of the hallmark indicators of the pathophysiology of cerebral ischemic injury. Glu is released into the extracellular space on the ipsilesional side of cerebral ischemia, with the highest concentrations detected in the later stages of Glu release [45]. GABA plays an important role in this process of functional reorganization of the motor cortex, in which pre-existing masking pathways are unmasked by reduced intracortical inhibition. Different components of the inhibitory signal may affect the excitation–inhibition balance and improve the motor rehabilitation correlation [46]. Acetylcholine (ACh) appears to function as a neuromodulator in the brain; however, it is the key excitatory neurotransmitter in the periphery. ACh crosses the synaptic gap and binds to ACh receptors that are tightly clustered on the surface of the muscle fibers, resulting in endplate potentials initiating muscle action potentials leading to muscle contraction. Research has shown that there is an initial release of monoamines in the ischemic brain, accompanied by a decrease in their synthesis [47]. 5-HT primarily mediates descending medial RST inputs to spinal motor neurons from the medial PMRF and is closely associated with spasticity. Serotonergic agents enhance spasticity, while anti-serotonergic agents promote the relaxation time of the spastic muscle. Moreover, the emergence of disinhibition of monoaminergic brainstem pathways following stroke leads to an increased serotonergic influence in the spinal cord. Previous research has established that the increased release of 5-HT from brainstem pathways after stroke leads to prolonged involuntary muscle activity and spasms [48]. A recent in vivo study showed that dopamine (DA) directly activates spinal motoneurons and can increase muscle tone by activating D1-like receptors on somatic motoneurons [49]. It has been demonstrated that the ipsilateral striatum releases large amounts of DA almost immediately after the occurrence of ischemic stroke [50]. DA may also indirectly promote motoneuron excitability by amplifying Gluergic transmission. Histamine inhibits the release of Glu and DA, possibly because of the activation of H3 receptors, which may inhibit neuronal excitability. Previous research has established that NE stimulates motor neurons to enhance muscle tone by amplifying Glu-driven excitation [51]. However, NE does not directly promote muscle tone by increasing spontaneous motor protein activity [52] or amplifying the effects of afferent inputs on motor neurons [53].

## 5. Therapeutic Interventions of PSS

### 5.1. Assessment

Motor recovery and compensation are different processes in patients following a stroke. To our knowledge, motor recovery is identified as the reappearance of the motor pattern with a similar pattern to before the central nervous system injury, whereas motor compensation is defined as the appearance of new motor patterns induced by the adaptation or replacement of remaining motor elements. From a functional recovery perspective, this recovery is characterized by compensation rather than full neurological restoration to a previous state, and impaired motor function can be improved by compensatory activities for dyskinesia [3]. It is not the case that improving spasticity can alleviate movement disorders associated with stroke; therefore, it is important to develop tailored treatments for different people. Moderate spasticity may be beneficial by reducing muscle atrophy, improving standing by providing muscle tone, providing cardiovascular benefits, and reducing the risk of deep vein thrombosis [54]. Treatment is warranted when PSS interferes with the quality or function of daily life, including problems with maintaining personal hygiene, dressing, writing, and socialization/interpersonal relationships [55]. Motor and sensory impairment, age, history of previous stroke, type of stroke, lesion location, lesion volume, and the course of disease progression are expected to be correlated with spasticity. Accordingly, it is important to collect complete clinical data, evaluate patients, and develop an appropriate therapeutic intervention. Individual measures of PSS and its complications are useful for facilitating early identification and intervention, especially for monitoring treatment outcomes. In the clinic, rating scales, questionnaires, neurophysiological measures, and instrumented test measurements were used to evaluate the severity of spasticity. However, no standardized functional tests and scales for PSS have been developed and validated specifically for this study.

### 5.2. The Recommended Approach

Pharmacological or surgical treatment is recommended when spasticity does not respond to education, physiotherapy, and nursing care, disrupts daily life activities and caregiving, and affects the quality of life. A recent study proposed oral medications, including baclofen, tizanidine, dantrolene, and benzodiazepines, to treat segmental and generalized spasticity [7]. The currently available data indicate that oral medications exhibit a limited effect on PSS and show significant dose-dependent adverse effects [8]. Baclofen is generally considered one of the most effective oral spasticity medications. When oral medications with baclofen or other treatments are ineffective or when adverse effects of treatment become intolerable, an intrathecal baclofen (ITB) delivery system is considered. Furthermore, ITB has been approved by the Food and Drug Administration (FDA) for the treatment of severe spasticity due to cerebral and spinal injuries. It was recommended that ITB can be considered as early as 3 to 6 months after stroke [56]. Baclofen inhibits the release of excitatory amino acids (Glu and aspartate) by stimulating γ aminobutyric acid receptors and exerting its antispastic effect. A program of combined ITB and physical therapy significantly reduced tension and stiffness and indicated short-term efficacy in relieving limb spasticity. However, the use of splints and tapes to prevent wrist and finger spasticity after stroke is not recommended. Neurolysis is commonly used for the treatment of spasticity, and nerve blocks using neurolytic agents (such as phenol and alcohol) have demonstrated efficacy in managing focal spasticity. Intrathecal phenol is considered an effective alternative drug for patients with spasticity unresponsive to other treatments. Axonotmesis might be the most likely factor in the reduction in spasticity following phenol blockade. The preferred treatment for PSS is botulinum toxin type A (BoNT-A) to release muscle tone, reduce spasticity-associated pain, improve gait, reduce malpositioning of limbs, and improve passive or active range of motion. The injection of BoNT-A before the spasticity becomes moderate or severe improves impairment and passive function. Moreover, early BoNT-A intervention can alter the natural evolution of PSS, and treatment outcomes appear to be more pronounced and longer lasting. BoNT-A reduces the release of ACh by combining with surface receptors on the presynaptic membranes of cholinergic nerve endings and relieves hypertonia. Although local intramuscular injection of BoNT-A is a safe and effective treatment for spasticity, it is costly [57]. Various interventions to reduce spasticity and improve functional outcomes after stroke have been addressed in the guidelines; however, the desired effect has not been achieved, and adverse effects remain intractable.

### 5.3. Progress in the Intervention of PSS

Recent studies reported research advances in strategies for PSS, as discussed below (Table 1).

#### 5.3.1. Neuroprotective Therapy

Cerebrolysin, a neuropeptide preparation, exhibits neuroprotective properties. Cerebrolysin has neurotrophic, anti-apoptotic, and anti-neuroinflammatory properties, leading to improved functional recovery in post-stroke rats [58,59,60]. The neuroprotective effects of cerebrolysin may be associated with neurobehavioral deficits and anxiety in mice [87]. The beneficial effect on mood and motivation was also proven in a case report [88]. The safety of cerebrolysin has been confirmed in a meta-analysis [89]. A daily intravenous infusion of 30 mL cerebrolysin or placebo administered for 10 consecutive days resulted in a significant initial improvement on the Modified Rankin Scale; however, the outcomes did not show enhancement at day 90 [90]. Conversely, patients treated with cerebrolysin (30 mL/day) once daily for 21 days, starting at 24 to 72 h post-stroke onset, remained stable and exhibited an improvement of three points on the NIH Stroke Scale (NIHSS) by day 90 [91]. Consequently, the efficacy of cerebrolysin in evaluating outcomes and long-term prognosis remains unclear, and more clinical studies are required to elucidate the precise mechanism and effects of cerebrolysin and identify the populations that will benefit the most.

Stem cell therapy, recognized as a potential neuro-regenerative therapy for stroke patients, has great potential for PSS [92]. A single dose of 1–2 × 10^6^ cells/kg allogenic umbilical cord–mesenchymal stem cells (UC-MSCs) administered intravenously post-stroke demonstrated improvements in patients’ upper extremity and muscle strength, spasticity, and fine motor functions [93]. It was indicated that bone marrow hematopoietic stem cell therapy significantly influenced upper extremity motor scores, daily life activities, somatosensory evoked potential scores, and motor evoked potential scores [94]. The paracrine action of MSCs may be the main condition for nerve repair with neurotrophic effects. Neuroprotective effects of MSCs to ameliorate spasticity may be related to angiogenesis and anti-inflammatory effects [61,62]. Treatment of chronic stroke patients with intrathecally and intravenously administered CD271 stem cells, at a dosage of 2–5 × 10^6^ cells/kg, followed by up to 12 months of monitoring, has been demonstrated to be an effective therapeutic approach [95]. Fever, headache, and stroke recurrence were the frequently reported adverse effects. However, there were no significant differences compared to controls [96]. The timing of administration, optimal dosage, and immune rejection are challenges for clinical application [97]. Although an intravenous injection does not ensure that the cells reach the target area, current evidence indicates that it is safe and effective [98]. Although there are variations, clinical trials of stem cell-based therapies remain in their early stages, and the inadequacy of the sample sizes included is not reflected in the metrics [96].

Molecular patterns associated with stroke and chronic neurological damage initiate the activation of the complement system, with the complement cascade being triggered early in the progression of brain injury [99]. Excessive activation of the complement system contributes to the development of secondary brain injury through a variety of intricate pathological mechanisms, including the production of numerous inflammatory mediators, increased cerebral edema, and enhanced tissue damage. Several drugs that inhibit all or part of the complement system, including C1 inhibitors (C1-INH), CVF, C3aR antagonists, small or large molecule C5aR antagonists, and sCD59, reduce ischemia–reperfusion brain injury [100]. Furthermore, intranasal administration of the C3a drug to wild-type mice, initiated 7 days post-stroke, accelerated motor function recovery and stimulated overall white matter reorganization, increased peri-infarct structural connectivity, and upregulated Igf1 and Thbs4 expression in the peri-infarct cortex [101]. There is also a potential link between intrathecal cholinergic activity and complement activation [102]. Despite the promising potential of complement inhibitors in the treatment of ischemic stroke, their clinical availability remains limited. Further research is required to elucidate the mechanisms and side effects of specific complement components in stroke pathology. Therefore, we hypothesize that, in the future, the complement system will be an effective treatment for PSS.

#### 5.3.2. Gene Therapy

A novel approach to the treatment of spasticity involves the regulation of neuromuscular overactivity through the delivery of genes that promote synaptic inhibition. Advances in targeting techniques now permit the selective transduction of specific cell populations, such as motor neurons. Intravenous gene therapy after middle cerebral artery occlusion (MCAO) protected BBB integrity, reduced neuroinflammation, increased neuronal density, improved survival after stroke, and improved behavioral performance [103]. Genes encoding glutamate decarboxylase, Tetanus Toxin Light Chain, and Kir2.1 have demonstrated the ability to inhibit both evoked and spontaneous neuronal activity in vitro [104]. Progress in gene transfer technology has facilitated the transfer of desired genetic material to human neurons; however, the development of safe, durable, controlled, and specific methods for gene delivery remains a significant obstacle in gene therapy. Numerous challenges complicate the clinical application of gene therapy in spasticity, necessitating meticulous and prolonged validation. Despite the lack of research on PSS, gene therapy continues to hold promise as a valuable tool for biological applications and research at a macro level.

#### 5.3.3. Targeted Therapy

The large molecular weight and poor penetration ability of the existing oral drugs for the treatment of spasticity make it difficult to target the brain through the BBB, and the influence of blood vessels in the ischemic penumbra makes it difficult for the drugs to reach the local area directly [105]. Smart drug delivery systems can react to internal and external stimuli to control drug release, enabling more personalized and adaptive therapies. Nanoparticles enhance treatment precision and reduce systemic side effects, meeting key post-stroke spasticity treatment needs. For example, nanomaterials facilitate the use of exosomes, a biomarker being developed for stroke diagnostics and a therapeutic agent that can cross the blood–brain barrier (BBB), to improve targeting and enhance efficacy. In addition, the recovery of motor function 24 hours later and again 14 days after cortical injury is significantly improved in monkeys treated with exosomes [106]. Despite the absence of clinical studies on PSS, several studies have established the efficacy of drug delivery systems in post-stroke rehabilitation and functional recovery. Scalability of production, regulatory hurdles, and considerations of cost-effectiveness are the main obstacles that must be addressed in order to realize the full potential in the clinical setting.

#### 5.3.4. Physiotherapy

Physiotherapy should prioritize a combination of active strategies over passive interventions.

##### Motor Therapy

Movement disorders caused by spasticity are characterized by significant coactivation of the antagonist muscles. Motor therapy for spasticity includes strength training, extremity ergometer training, static stretching, dynamic stretching, positional orthoses, and constraint-induced movement therapy (CIMT). Strength training was superior or at least equal to conventional therapy, other therapies, or no intervention in improving spasticity as well as function, strength, gait, and balance [63]. The experimental group underwent conventional physiotherapy and unilateral strength training targeting the less affected wrist extensor muscles over a 4-week period, consisting of twelve sessions at a frequency of three sessions per week. The combination of unilateral strength training and conventional physiotherapy appears to be improving strength, motor function, and spasticity in stroke patients, which may be induced through cortical excitability [64]. Notably, significant reductions in EMG position threshold and burst duration were observed at higher speeds (≥120°/s) (*p* < 0.05), along with a decrease in passive torque following washout in patients with chronic hemiplegia who engaged in functional task practice. High-intensity dynamic resistance training improved stretch reflex regulation and enhanced neuromuscular activation compared to functional task practice, with no deleterious consequences [107]. Stretching muscles has become a standard spasticity rehabilitation approach [108]. Prolonged passive static stretching is a widely used technique recommended to reduce spasticity after stroke [109]. The two primary objectives of stretching are enhancing the viscoelasticity of musculotendinous units and lowering the excitability of the motor neurons. Furthermore, patients who participated in functional stretching therapy thrice weekly for four weeks exhibited significant modifications in the neural and mechanical properties of the spastic medial gastrocnemius muscle, with these improvements persisting at a two-month follow-up [65]. Furthermore, stretching is frequently used with other adjunctive therapies, including casts, splints, and orthotics. The role played by resistance movements in spasticity treatment requires further research. Additionally, individuals who performed pedaling exercises using a pedaling dynamometer set at a load of 5 Nm and a speed of 40 rpm demonstrated a significant increase in the flexion angle and angular velocity of the knee and hip joints immediately after a 10 min intervention [110]. Static stretching with positioning orthoses reduces post-stroke wrist flexion spasticity; however, static stretching with simple positioning is not superior to traditional physiotherapy for joint mobility [111]. Although using splints is controversial for preventing spasticity in spasticity clinics, using hand splints to reduce spasticity in the wrist and fingers is not recommended [112]. CIMT is effective for spasticity and motor impairment in the upper [113] and lower [114] extremities. Restraining the movement of the healthy limb by splinting or slinging, enforcing the use of the affected limb for movement, and applying molding methods to massively practice the affected limb have been proposed as useful tools for recovering abilities in everyday activities [115]. CIMT is only used in a small percentage of paralyzed patients and is not suitable for patients with severe impairment or those in the first phase of rehabilitation training [116]. The modified CIMT significantly improved spasticity and motor function scales compared to physiotherapy [66]. After 23 years, a patient with massive right frontoparietal infarction and completely nonfunctional left-hand spasticity exhibited widespread neural activation on fMRI, reduction in spasticity, and significant improvement in function due to persistent swimming and the use of SaeboGlove [117]. Motor therapy has been shown to reduce spasticity beyond the generally accepted recovery period.

##### Peripheral Sensory Stimulation

Neurostimulation techniques are ideally suited for use during stroke recovery due to their capacity to target specific anatomical structures or neuronal networks, as well as precise timing and dosage [118]. Repetitive transcranial magnetic stimulation (rTMS) can be used to treat PSS and reduce hypertonia [119]. Patients with unilateral stroke in the middle cerebral artery region who received inhibitory and low-frequency rTMS in the contralateral motor cortex increased connectivity with the left angular gyrus [67], which indicated stimulation of neuronal plasticity processes. rTMS reduces PSS; however, the mechanisms through which rTMS might influence spasticity are also not understood. Contralateral motor cortex 1 Hz rTMS combined with physiotherapy reduced the stretch reflex-mediated component of muscle stretch resistance and reduced spinal excitability [120,121]. Functional electrical stimulation has been widely used in the neurorehabilitation of individuals with paralysis due to spinal cord injuries or stroke aftereffects [122]. Transcutaneous electrical nerve stimulation (TENS) administered at 200 Hz for 30 min effectively reduced lower extremity spasticity in chronic stroke survivors when combined with other sensory inputs and task-specific exercises [68]. In 2021, the FDA approved vagus nerve stimulation as an adjunct to intensive rehabilitation for the treatment of chronic upper extremity dysfunction following ischemic stroke. It has been discovered that localized muscle vibration therapy (VT) is safe and viable and is frequently incorporated into routine neurorehabilitation to improve spasticity in post-stroke patients [123]. Brief 15–20 min sessions of vibration training at a frequency of 40 Hz, in conjunction with physical therapy, significantly enhanced the remission rate among patients with Motor Apraxia Syndrome (MAS) [69]. Moreover, VT significantly reduced pain and improved walking by altering muscle stiffness in patients with PSS [124]. However, VT exhibited a pronounced antispasticity effect in the upper limbs and a limited effect in the lower limbs due to the distribution of spasticity [125]. Extracorporeal shock wave therapy (ESWT) exhibits a dose–response effectiveness on rehabilitation in stroke patients, including pain relief, reduction in muscle spasticity, increased control, and enhancement of functional mobility in both the upper and lower extremities [70,126]. The therapeutic effect may vary depending on the region of application of ESWT, which is applied simultaneously to both the muscle–tendon junction and the middle part of the muscle for the best treatment effect [71]. ESWT may improve spasticity by reducing motor neuron excitability [72] and improving muscle properties [73]. Telerehabilitation, which has become a crucial aspect of post-stroke care during and after the COVID-19 pandemic, was shown to be cost-effective and improve rehabilitation adherence. Following discharge from the hospital, patients commenced telerehabilitation sessions with their physical therapists, guided by a customized rehabilitation exercise program. They received appropriate guidance on conducting clinical and virtual evaluations during the treatment period. Telerehabilitation and tele-evaluation are safe and have long-term efficacy in enhancing the effects of conventional physiotherapy and rESWT on post-stroke spasticity [127].

##### Bioelectronic Medicine

The neuroscience discoveries form the foundation needed to understand the emergence of new technologies such as robotics and virtual reality (VR), which provide repetitive and final motion even in severely impaired patients. The degree of equivalence between robotic training (10 sessions lasting 60 min each, 2 or 3 days a week) and botulinum toxin regarding motor recovery and spasticity reduction [128] has been examined. Robotic therapy combined with botulinum toxin injections can improve spasticity, and robotic training (45 min/session, two sessions/week) can improve muscle strength [74]. Moreover, the efficacy of robotic therapy may depend on various factors, including training intensity, training time, type of therapy, robotic equipment, and patient characteristics. It can help physicians and therapists better face the challenges of technical neurorehabilitation by providing movement control and measurement reliability [129]. Patients are tasked with using a computerized VR system to perform active or passive movements based on multisensory feedback using the paralyzed limb [130]. Repetitive goal-directed tasks are performed in VR-based interventions to induce dependent neuroplasticity, thereby improving motor recovery and cognition. Patients engaged in VR therapy for 60 min daily, five times per week, over a four-week period. As a result, the altered activations subsided, and there was a predominant activation of the ipsilesional SM1. This was specifically demonstrated by the reorganization of motor representations in the M1, PM, SMA, and somatosensory cortex due to synaptic efficacy and dendritic spine remodeling [131]. A single-blind, randomized trial demonstrated that the combination of VR and real instrument training (30 min per day, 3 days a week, for 6 weeks) significantly improved elbow spasticity and promoted the recovery of upper-limb and cognitive functions [75]. In summary, VR offers unique advantages for treating spasticity with cognitive impairment after a stroke.

##### Biofeedback Therapy

Biofeedback (BFB) therapy is a common rehabilitation strategy that increases corticospinal excitability and expands motor cortical representations of stimulated body regions by combining motor training with sensory information. Compared to conventional treatment, patients received 6 weeks of myofeedback training twice a week, which significantly improved spasticity in patients with PSS [132]. EMG-BFB therapy assists patients in understanding the limb muscle motor activation location by translating the patient’s physiological electrical response into aural or visual stimuli, thus improving awareness of new target muscles, which in turn facilitates separation of muscle activity and provides valuable feedback to the patient without activating the muscle. It helps the patient establish a new movement pattern of cognitive demand processes and guides the patient to use the new movement pattern [76]. Mirror therapy (MT), a therapeutic approach similar to BFB, stimulates the motor cortex through constant visual feedback, affecting the electrical activity and excitability of the cortex, promoting brain function remodeling, and inducing motor function recovery [133]. MT significantly improved limb motor function and pain perception compared to conventional therapy [77]. For stroke patients who receive BoNT-A injections, subsequent MT training for 5 days/week, 6 h/day, over 3 weeks effectively improves motor function and daily activities and reduces spasticity [134].

#### 5.3.5. NexTGen Therapy

Brain–computer interfaces (BCIs) are systems that can acquire and analyze brain signals and convert them into commands that are relayed to output devices to perform desired operations. BCIs based on functional electrical stimulation have been used for motor rehabilitation of the upper limbs after stroke and for improvement of hand and finger spasticity after therapy [78]. The concept underpinning Neuralink involves the development of a brain–machine interface aimed at restoring sensory and motor functions in patients with neurological disorders. The device employs “threads”, which are minuscule electrodes surgically implanted into the brain using a robot specifically designed by the Neuralink team. These electrodes facilitate the collection of real-time data on the activity of selected neuronal groups, which are algorithmically processed and stored in an external Neuralink device. These data can be accessed via the Neuralink iPhone application. Notably, Neuralink testers have demonstrated the ability to manipulate a cursor on a screen solely through thought [135]. The recent FDA approval for Neuralink’s clinical human trials represents a significant advancement. Nonetheless, considerations regarding safety and ethics remain paramount [136]. The current paucity of data necessitates further investigation and research. Additionally, clinical trials are essential for Neuralink’s acceptance and integration into the future of neurosurgical practice. This also suggests a transformative approach that advocates for the integration of alternative methods to traditional animal testing, including cell-based assays using human induced pluripotent stem cell-derived organoids and organ-on-a-chip technology, combined with sophisticated artificial intelligence methods [137].

#### 5.3.6. Complementary and Alternative Medicine

##### Traditional Chinese Medicine Formulas

Traditional Chinese medicine (TCM) formulas have been widely used clinically as a potential therapeutic strategy for the recovery of stroke and sensorimotor functions. Previous research has expressed that combining oral and topical treatment is beneficial for reducing spasticity in both the upper and lower extremities. Furthermore, both were considered well-tolerated potential therapies with mild self-healing adverse events [138]. On the one hand, SYGCD and GLGZD could regulate the balance of the neurotransmitter system, including the increase in the concentration of inhibitory amino acids and the expression of their receptors, and the decrease in excitatory amino acids, thereby improving the spastic paralysis after stroke [79,139]. On the other hand, SYGCD [80] and GLGZD [81] improve neurological deficits and survival of infarcted cortical neurons by negatively regulating inflammation. Moreover, GLGZD attenuates neuronal injury and reduces infarct volume by inhibiting neuronal apoptosis and enhancing angiogenesis [140,141]. BSLSD [142] and BYHWD [82] ameliorated synaptic plasticity in peri-infarct brain tissue. Additionally, BYHWD could mitigate oxidative damage and improve neurogenesis in MCAO-R rats [83,143]. The effective delivery of TCM is significantly impeded by the disruption of the BBB structure and increased permeability after stroke. The lack of targeting and low bioavailability makes it difficult to accumulate and maintain an effective therapeutic concentration in the lesion area even upon entry into the brain. The delivery, targeting, and therapeutic effects of formulas require further research.

##### Acupuncture

The positive effects of acupuncture on PSS are clinically recognized in 11 countries [144]. Electroacupuncture (EA) relieves spasticity by decreasing the inflammatory response and inducing the gut–brain axis [145]. It has been proven that EA promotes the expression of angiogenic factors and inhibits the production of the anti-angiogenic factor endothelial depressor [84]. Additionally, studies indicate that EA increases cerebral blood flow on the affected side by releasing ACh and decreasing the expression of angiotensin II and its type 1 receptor [146,147]. An animal study suggested that acupuncture could significantly decrease spinal hyperreflexia and muscle tone by activating the KCC2-mediated GABA signaling pathway in the spinal cord [85]. Moreover, waggle needling could probably decrease spasticity by enhancing inhibitory neurotransmitter concentration in the brainstem, thereby decreasing the proportion of type I muscles [148]. Acupuncture has previously been reported to improve limb function in chronic hemiplegic stroke patients by increasing the activity of the motor cortex on the affected side and the activity of brain regions related to sensory integration and motor coordination [149,150]. Dry needling, fire needling, and functional needling have beneficial clinical efficacy in alleviating spasticity, promoting motor function recovery, and improving the ability to perform daily life activities, but their mechanisms of action have not yet been clarified [86,151,152]. Although the efficacy and safety of acupuncture in treating PSS remain uncertain due to limitations and inconsistencies in the available evidence, it is a promising complementary treatment for PSS, and the antispasmodic effect increases with more sessions [153].

### 5.4. Limitations

There were several limitations in our study. Firstly, we did not conduct further subgroup analyses of trials with separate acute, chronic, and recurrent strokes due to insufficient data, and different stroke types may have influenced clinical decision-making. Secondly, age, history of previous stroke, lesion site, and lesion volume were discussed vigorously in the intervention studies; however, the proportion of these factors varied between the intervention and control groups in all included studies. Thirdly, since most of the studies on complementary alternative therapies have been conducted in China, ethnic differences may limit the extrapolation of our findings to some extent. Fourthly, some interventions, such as motor therapy and traditional Chinese medicine formulas, lack large and robust clinical trials. Future larger randomized clinical trials oriented to PSS and more homogeneous characteristics of participants from around the world are needed to demonstrate the efficacy of potential strategies to improve PSS.

## 6. Conclusions

Spasticity, the most common post-stroke complication, can disrupt motor recovery and affect daily life. Spasticity is unlikely to be caused by a single mechanism; rather, it is a complex chain of changes in several interdependent networks. Understanding the phenomenon of spasticity is necessary to develop medications and therapeutic strategies that can effectively treat the cause rather than the symptoms. Exploring the complex mechanisms underlying the onset of spasticity through measurement and assessment could increase the possibilities of developing a more optimal and differentiated approach to neurorehabilitation. Neuroimaging studies highlight the importance of some specific brain areas (e.g., SMA, CMA, and secondary somatosensory cortex) in the development of spasticity and other motor disorders. Nevertheless, these motor issues are not well understood and further neuroimaging studies, which allow for non-invasive investigation of brain dysfunction without patient discomfort, should be conducted to monitor the onset and development of spasticity. Several interventions with segmental and generalized spasticity-improving effects have been recently identified; however, most of them remain in animal experiments or phase I clinical stages. Despite the scarcity of clinical trial data, suggested interventions and treatment objectives need to account for the influence of neuropsychological, cognitive, and behavioral deficits on the recovery process. In addition, large, rigorously studied, and appropriately controlled trials are necessary to improve the management of PSS. Stratified clinical trials and individualized treatment trials require large numbers of patients, which can only be achieved through multicenter clinical trials and standardized diagnostic criteria and outcome measures. With increasing attention to the prognosis of stroke, more advanced and targeted treatment strategies are anticipated to be developed and implemented to improve patients’ efficacy and quality of life.

## Figures and Tables

**Table 1:** Research advances in strategies for PSS.

Intervention Strategies	Intervention Given Outcomes	Potential Mechanisms	Advantages	Disadvantages	References
Cerebrolysin	Increases Tuj1 and CNPase; Inhibits calpain and reduces the number of apoptotic cells; Increases TNF-α, IL-1β, iNOS, CD206, and YM1/2.	Neurogenesis; Inhibits apoptosis; Inhibits neuroinflammation.	Easy to use; Accessible; Affordable; Safe; Emotional benefits.	Long-term prognosis remains unclear.	[58,59,60]
Stem cell therapy	Increases Nestin, Ki-7, DCX, and CD31; Increases GAP-43 and IL 10 and decreases IL-1β, TNF-α, and IL-6.	Angiogenic; Anti-inflammatory.	Safe; Effective; Feasible.	Uncertain timing of administration, optimal dose, and immune rejection.	[61,62]
Strength training	Greater MEP amplitude and shorter CSP duration in the ipsiH; Increases maximal isometric joint torque, agonist EMG, and peak power.	Improves cortical excitability; Improves stretch reflex modulation.	Feasibility and safety; Efficacy; Personalized and thorough strategies.	High costs.	[63,64]
Dynamic stretching	Increases the pennation angle and Hmax/Mmax ratio; Decreases H-reflex latency.	Activates mechanotransduction.	Improves activities of daily life.	Limited effect.	[65]
Constraint-induced movement therapy	Log-28.	Cortical reorganization.	Improves proprioception, motor function, and activities of daily life.	Lack of long-term follow-up assessment.	[66]
Repetitive transcranial magnetic stimulation	Increases connectivity to the left angular gyrus.	Neuronal plasticity.	Effective; Feasible.	Lack of optimal treatment protocol.	[67]
Transcutaneous electrical stimulation	Improves presynaptic inhibition.	Synaptic plasticity.	Low cost, comfort of use, and absence of adverse reactions.	Uncertain frequency and long duration.	[68]
Vibration therapy	Decreases ultrasound variables of the paretic medial gastrocnemius.	Alters muscle stiffness.	Effective; Well tolerated.	Lack of long-lasting effects.	[69]
Extracorporeal shock wave therapy	Decreases the Hmax/Mmax ratio; Increases muscle echo intensity.	Improves α motor neuron excitability; Improves muscle properties.	Dose–response effectiveness.	Uncertain frequency, duration, and intensity.	[70,71,72,73]
Robotic training	Decreases co-contractions; Increases agonist muscles in sEMG.	Alters muscle stiffness.	Efficacy.	Small sample; High cost.	[74]
Virtual reality	Increases ipsilesional activation at the SM1 area in MRI.	Neuroplastic.	Safe; Lower cost; Feasible.	Small sample.	[75]
Electromyographic biofeedback	Decreases TSRT.	Establishment of new movement patterns.	Increase in mobility.	Small sample.	[76]
Mirror therapy	A shift in activation balance M1 toward the lesioned hemisphere in fMRI.	Cortical reorganization.	Improves limb motor function and pain.	Small sample.	[77]
Brain–computer interfaces	Decrease MAS.	Provide proprioceptive feedback.	Improvement in life.	Heterogenous with respect to stroke type and lesion location; Small sample.	[78]
Shaoyao Gancao Decoction	Increases the content of inhibitory amino acids and the expression of their receptors, and decreases excitatory amino acids; Decreases the expression of IL-1β, TNF-α, and MCP-1, while increasing the number of IL-10.	Regulates the balance of the neurotransmitter system; Inhibits the activation of microglia and astrocytes.	Well tolerated; Ameliorates neurological deficits.	Mechanisms remain unclear.	[79,80]
Gualou Guizhi Decoction	Reduces Glu, Asp, and Gly levels in the cerebrospinal fluid.	Modulates excitatory amino acids.	Easy administration and few adverse reactions.	Dosage and mechanism are unclear.	[81]
Baishao Luoshi Decoction	Decreases MAS and mNSS; Rescues synaptic structure.	Rescues synaptic plasticity.	Few adverse reactions.	Mechanism is unclear.	[82]
Buyang Huanwu Decoction	Increases SYN, GAP-43, and MAP-2.	Facilitates neurorehabilitation through synaptic plasticity.	Effective; Well tolerated.	Failure to assess temporal variability.	[83]
Electroacupuncture	Increases IL-6, TNF-α, and TMAO, downregulates NF-κB p65, NLRP3, caspase3, and caspase9, and increases n-propyl acetate and propyl butyrate levels.	Decreases the inflammatory response and modulates the gut–brain axis.	Activates the endogenous self-protection mechanism.	Small sample; brain tissue is not well collected.	[84]
Waggle needling	Enhances GABA, KCC2, and GABAAγ2.	Decreases the proportion of type I muscle.	Stronger stimulation and superior antispastic effect.	Complete conduction pathway remains unclear.	[85]
Dry needling	Increases directional control backward and affects backward direction.	Alters muscle stiffness.	Improves balance, range of motion, and accuracy of maintaining stability.	Mechanism is unclear.	[86]

The abbreviations used are defined as follows: IS, ischemic stroke; HS, hemorrhagic stroke; CIRI, cerebral ischemia–reperfusion injury; CBF, cerebral blood flow; Tuj1, class III β-tubulin; CNP ase, 2′;3′-cyclic nucleotide 3′-phosphodiesterase; TNF-α, tumor necrosis factor-α; IL-1β, interleukin-1β; iNOS, inducible nitric oxide synthase; CD206, cluster of differentiation 206; YM1/2, chitinase-like proteins; CREB, cAMP response element-binding protein; PGC-1α, peroxisome proliferator-activated receptor gamma coactivator 1α; DCX, doublecortin; CD31, platelet endothelial cell adhesion molecule-1; GAP-43, growth associated protein-43; IL 10, interleukin-10; IL-6, interleukin-6; EMG, electromyogram; MRI, magnetic resonance imaging; TSRT, template-switching reverse transcription; mNSS, modified neurological severity score; SM1, primary sensorimotor cortex; fMRI, functional magnetic resonance imaging; MCP-1, monocyte chemoattractant protein-1; Glu, glutamate; Asp, aspartic acid; Gly, glycine; TGF-β, transforming growth factor-β; NO, nitric oxide; eNOS, endothelial nitric oxide synthase; VEGF, vascular endothelial growth factor; VEGFR, vascular endothelial growth factor receptor; MAS, assessment scale; mNSS, modified neurological severity scores; SYN, synaptophysin; GAP-43, growth associated protein-43; MAP-2, microtubule-associated protein-2; DCX, doublecortin; BDNF, brain-derived neurotrophic factor; ROS, reactive oxygen species; MDA, malondialdehyde; 8-OHdG, 8-hydroxy 2′-deoxyguanosine; TMAO, trimethylamine N-oxide; NF-κB, nuclear factor kappa-B; NLRP3, nod-like receptor protein-3; mAChR, muscarinic acetylcholine receptor; AT2R, angiotensin II type 2 receptor; Gq, G protein q subunit; CaM, calmodulin; GABA, gamma-aminobutyric acid; KCC2, neuron-specific K-Cl Cotransporter; VMHC, voxel-mirrored homotopic connectivity; ReHo, regional homogeneity.
